# Nanoparticle-Based Therapies for Management of Subarachnoid Hemorrhage, Neurotrauma, and Stroke

**DOI:** 10.3390/biomedicines13010016

**Published:** 2024-12-26

**Authors:** Grace Hey, Ilyas Mehkri, Yusuf Mehkri, Hasan Maqbool, Mubariz Tahirkheli, Samuel Woodford, Brandon Lucke-Wold

**Affiliations:** 1 College of Medicine, University of Florida, Gainesville, FL 32610, USA; gracehey@ufl.edu (G.H.); ymehkri@ufl.edu (Y.M.);; 2 Lillian S. Wells Department of Neurosurgery, College of Medicine, University of Florida, 1600 SW Archer Road, Gainesville, FL 32610, USA

**Keywords:** nanoparticle, neurotrauma, stroke, subarachnoid hemorrhage

## Abstract

Neurotrauma, stroke, and subarachnoid hemorrhage (SAH) are symptomatically diverse and etiologically complex central nervous system pathologies. Despite numerous therapeutic modalities that are available to minimize neurologic damage and secondary injury, the prognosis can still be dismal and unpredictable. Nanoparticle (NP) technology allows for deliberate, modular, and minimally invasive drug delivery. This literature review encompasses pertinent information on the impact and versatility of nanoparticle therapeutics when treating neurotrauma, stroke, and SAH. Currently, notable treatments such as Perfluorooctyl-Bromide (PFOB), PLGA nanoparticles, and ischemic relief-based NPs are promising new techniques for the management of these complex pathologies.

## 1. Introduction

Due to their size and multifunctionality, nanoparticles (NPs) have emerged as a promising new method for targeted drug delivery. Nanoparticles convey several advantages, including their ability to carry drug payloads, deliberate drug release, and modification of the overall pharmacokinetics of drugs [1]. Conveniently, they can be administered by a variety of routes, including consumption and inhalation [2]. In the realm of neurosurgery, the highly restrictive nature of the blood–brain barrier (BBB) presents significant challenges in optimal drug delivery [3]. However, the lipophilicity and size of nanoparticles promote the passage of pharmaceuticals across the BBB, effectively allowing for the delivery of targeted drugs that were once unable to enter the brain. Nanoparticles additionally can increase the concentration of drugs adjacent to the BBB, thereby increasing the probability that a drug will cross the BBB.

**Figure 1:**
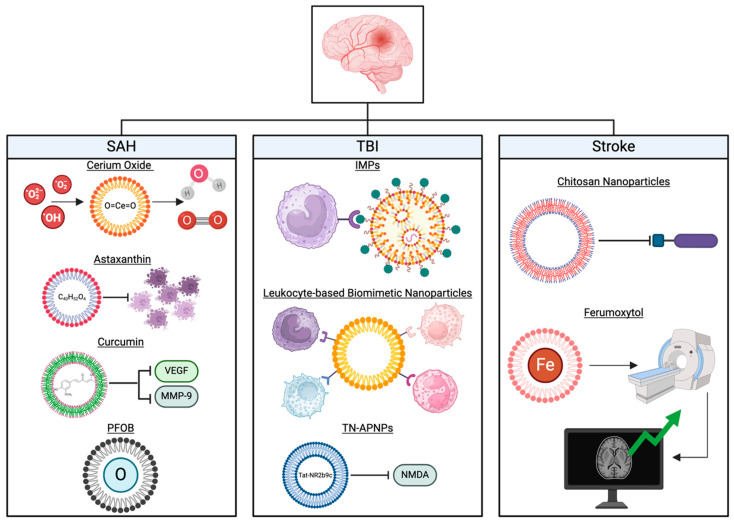
Overview of nanoparticle therapies for SAH, TBI, and stroke. SAH: The mechanism of action of cerium oxide is similar to that of superoxide dismutase due to its primary action of ROS scavenging. Astaxanthin also reduces ROS but mainly through the inhibition of apoptosis. Curcumin can suppress the actions of VEGF and MMP-9. PFOB has a high oxygen-carrying capacity and has been proposed to work by supplying oxygen to tissues in need and removing excess carbon dioxide from the site of the lesion. TBI: PLGA is a biodegradable and biocompatible polymer that can be used to construct nanoparticles that contain compounds used for the treatment of TBI. IMPs can be encapsulated in PGLA to remove hematogenous monocytes by binding to macrophage receptors following TBI. Leukocyte-based biomimetic nanoparticles mimic leukocytes and target leukocyte-specific inflammation. TN-APNPs transport the Tat-NR2b9c peptide, which targets NMDA-induced neurotoxicity. Stroke: Nanoparticles for stroke have largely been studied as a delivery system for anti-inflammatory compounds and an agent to enhance neuroimaging. Chitosan nanoparticles work by inhibiting caspase-3, a pro-inflammatory compound related to stroke pathogenesis. Ferumoxytol is an iron oxide nanoparticle that can be used to improve sensitivity and contrast enhancement in MRI scans.

There exists a variety of NPs, including lipid-based NPs, solid lipid NPs (SLNs), and polymeric NPs. Liposomes are vesicular bilayers composed of amphiphilic lipids. Notably, liposomes were the first NPs tested for drug delivery [4]. SLNs exhibit a solid hydrophobic core in which the drug can be dissolved. Importantly, SLNs are usually able to cross the tight endothelial cells of the BBB [5,6]. Polymeric NPs are composed of a core polymer matrix in which drugs are embedded [7]. These polymers have been designed for medical applications and frequently include polylactides (PLAs), polyglycolides (PGAs), and poly(lactide-co-glycolides) (PLGAs). Herein, we evaluate the diverse range of NPs currently being examined for their efficacy as therapeutic agents in circulation, their deliberate release to target sites, their enhancement of BBB permeability, and their obviation of the reticuloendothelial system while still maintaining positive patient outcomes (Figure 1).

## 2. Subarachnoid Hemorrhage

Subarachnoid hemorrhage (SAH) is a medical emergency characterized by acute bleeding in the subarachnoid space [8]. The onset of intracranial aneurysm rupture leads to early brain injury (EBI), and if left untreated, patients may experience increased intracranial pressure, cerebral ischemia, BBB disruption, and sudden neuronal apoptosis within 72 h [9]. SAH conveys significant morbidity and mortality rates worldwide and, as such, highlights the necessity to develop novel therapeutic strategies [10]. Nanoparticle use for SAH remains largely in the preclinical stage, although recent evidence suggests that targeting the specific mechanisms of EBI could be an effective treatment [9,11].

### 2.1. Cerium Oxide Nanoparticles in the Scavenging of Reactive Oxygenated Species

Following aneurysmal SAH, cerebral ischemia and free-radical formation are known sequelae [12]. Extracellular hemoglobin from blood leakage undergoes increased autoxidation and subsequently forms reactive oxygenated species (ROS) [10]. The presence of ROS following SAH results in significant neuroinflammation. Specifically, pathological changes in vasculature morphology and increased levels of pro-inflammatory cytokines—including IL-1, IL-6, and TNF-a—recruit neutrophils, monocytes, and lymphocytes to the site of neurologic injury, which exacerbate inflammation [13]. As such, preventing the formation of ROS and subsequent inflammation can help in the amelioration of SAH.

Cerium oxide nanoparticles (CeNPs) can switch between +2 and +3 oxidation states and cause an autocatalytic, positive feedback loop to scavenge ROS [14]. Cerium oxide displays antioxidant properties similar to those of superoxide dismutase, a key enzyme in human metabolism responsible for reakdown of ROS [15]. The antioxidant properties of CeNPs have led to the investigation of their applications in Alzheimer’s disease, Parkinson’s disease, multiple sclerosis, ischemic stroke, and amyotrophic lateral sclerosis [16]. In a murine model of cerebral ischemia, CeNPs have been shown to reduce ischemic cell death by approximately 50% [17]. Later work by Kim et al. [18] demonstrated significant neuroprotective effects against ROS-induced cell death in mice treated with CeNPs. More recently, CeNPs have been shown to significantly reduce neuronal death, macrophage infiltration, and brain edema in a murine model of SAH [19]. Here, CeNP-injected rats demonstrated significantly increased survival rates of 88.2% compared to controls.

Similarly, nuclear factor-erythroid 2 p45-related factor 2 (Nrf2) is upregulated following SAH in rats and plays a role in reducing edema, apoptosis of nerve cells, and destruction of the BBB [20,21]. Nrf2 is a transcription factor widely expressed in the central nervous system that upregulates the expression of genes responsible for the generation of antioxidants [22]. Molecules such as melatonin, curcumin, and erythropoietin work to activate Nrf2 and, as such, have the potential to improve neuroinflammation through ROS scavenging following SAH. Superoxide dismutase, glutathione peroxidase, and catalase work to scavenge ROS in the central nervous system and are significantly reduced following SAH [22].

Currently, there exist no reports in the literature regarding the use of CeNPs, Nrf2, superoxide dismutase, glutathione, peroxidase, or catalase NPs for SAH in humans. However, given the survival benefits and reduction in neuroinflammation seen in rodent models of ROS, it can be hypothesized that CeNPs have the potential to elicit similar effects in human subjects. Careful consideration and significant research efforts are necessary to determine optimal formulations, dosages, and delivery methods to determine potential drug interactions and avoid acute and chronic toxicity such as cytokine storm, immune dysregulation, and organ damage.

### 2.2. Antioxidant and Anti-Inflammatory Properties of Astaxanthin

The astaxanthin (ATX) nanoparticle is a common yet powerful carotenoid antioxidant [12]. ATX has been shown to suppress ischemic brain injuries by the mitigation of ROS-induced apoptosis [19,23]. One drawback to the clinical use of ATX is its low solubility with intravenous and oral administration [19,24]. This presents a significant challenge to clinicians, as drugs are frequently administered orally or intravenously. However, You et al. developed a strategy to load ATX onto *Fe*_3_*O*_4_ (transferrin) in a polyethylene glycol layer to reach a specific BBB receptor target, thereby avoiding solubility and instability concerns [25]. Here, Fe_3_O_4_-bound ATX loaded in polyethylene glycol was shown to significantly reduce cellular apoptosis [25]. It is important to note that contradictory reports exist in the literature surrounding the efficacy of ATX in vivo. Cai et al. demonstrated that Fe_3_O_4_-bound ATX demonstrates incomplete effectiveness due to the insufficient release of ATX from the polyethylene glycol protective layer [25]. The challenge of ATX release presents a barrier to clinical use, as ATX must be released from the polyethylene glycol to have beneficial effects on the body. Strategies to improve polyethylene glycol release include modifying the concentration, pH, hydrophilic/hydrophobic balance, and temperature sensitivity of polyethylene glycol. Other researchers have utilized direct ultrasound focusing to deliver ATX directly to the central nervous system [26]. Cai et al. [27] investigated this strategy by injecting SAH mice with ATX NPs divided into three activation groups: general release, pH response release, and ultrasound-triggered release. Ultrasound-focused ATX release was found to have good biosafety and improved overall survival rates when compared with control [27]. However, the use of ATX NPs for SAH is fairly novel and remains largely in the preclinical phase. Additionally, animal studies are necessary to optimize ATX dosing and delivery methods before human clinical trials to ensure patient safety.

### 2.3. Neuroprotective Effects of Curcumin After BBB Disruption

Curcumin nanoparticles (C-NPs), much like ATX and CeNPs, have strong antioxidant, anti-inflammatory, and anti-apoptosis characteristics [24]. C-NPs demonstrate minuscule circulation in the body and can easily pass through the BBB [28]. C-NPs injected intraperitoneally have been shown to suppress vascular endothelial growth factor (VEGF) activity in rats, effectively disrupting the BBB and preventing disruption through the survival of key tight junction proteins [27]. The retention of C-NPs in the circulatory system and brain can be extended by PLGA encapsulation [28]. Furthermore, MMP-9 expression has been proven to cause EBI after SAH with its role in the formation of cerebral edemas [29]. C-NPs have been shown to preserve the BBB through MMP-9 suppression [30,31]. The extent of C-NP remains largely in the preclinical stages as of now.

### 2.4. Early Brain Injury Mitigation Effects of Perfluorooctyl-Bromide (PFOB)

PFOB is a type of perfluorocarbon (PFC) that carries oxygen through emulsification [32]. When used in combination with *Fe*_3_*O*_4_ nanoparticles, PFOB can be used as a contrast agent in US, CT, and MR imaging. Using PFOB for multimodality imaging may allow physicians to increase the accuracy of their diagnoses [33]. PFOB exhibits a high oxygen-carrying capacity and an ability to rapidly act on lesions, thereby providing a neuroprotective effect to mitigate an SAH-induced EBI [34]. Two distinct mechanisms of protection have been proposed: (1) the high oxygen-carrying capacity of PFOB can resupply oxygen to tissues in need, and (2) PFOB can remove excess carbon dioxide to decrease viscosity and increase blood flow. Additionally, these mechanisms contribute to reduced free-radical formation to protect against further neuronal apoptosis and increased EBI [14]. It has been proposed that Hypoxia-Inducible Factor-1 alpha (HIF-1α) may play a role in this biological pathway [35]. In the context of hypoxia, HIF-1α expression downregulates transcriptions of genes that maintain ATP production [32]. It has been proposed that the inhibition of HIF-1α inhibits downstream genes activated during EBI, which promote neuronal apoptosis; however, the PFOB mechanisms of EBI amelioration are not specific to HIF-1α regulation [32]. The lack of supportive literature on the use and action of PFOB nanoparticles suggests that this is still an area of early investigation.

While there is evidence of PFC as a protective agent in other ischemic injuries [36], the literature on its use in SAH is sparse. To our knowledge, no studies testing drug conjugation to PFOBs for the treatment of SAH have been conducted. However, the utilization of PFOBs as a drug delivery system to the site of an acute hemorrhage could prove invaluable, just as other PFC NPs have been conjugated to specific drugs such as urokinase and fumagillin to promote thrombolysis and antiangiogenesis, respectively [37]. Future studies regarding BBB penetration, effects on neurons, and overall metabolic interactions should be investigated to determine the potential efficacy and safety of this therapy for SAH.

## 3. Neurotrauma

Traumatic brain injury (TBI) is a leading cause of death globally [33]. The initial injury, referred to as the primary injury, results from a mechanical force directed to the brain. This force causes damage by shearing, tearing, or stretching neurons, axons, glia, and blood vessels, often those that comprise the BBB [38]. The downstream effects of the initial injury promote neuroinflammation, excitotoxicity, and further BBB disruption, which is known as the secondary injury [39,40]. This occurs as a result of the production of toxic and pro-inflammatory molecules such as prostaglandins and oxidative metabolites [38], which allows for the extravasation of plasma proteins, monocytes, macrophages, and leukocytes across the BBB as well as further neurotoxicity [41,42,43]. Neuroinflammation also involves the release of inflammatory cytokines and the activation of microglia [44]. Ultimately, the treatment of TBI must repair neurofunction by addressing both the primary injury and reducing the impact of the neuroinflammation-induced secondary injury [38]. The BBB has been shown to be disrupted within the first twenty-four hours following a TBI, providing a potential therapeutic window for nanoparticle technology, as several tested NPs have successfully reached the brain following this injury [45]. In this discussion, we examine the various types of NPs, their surface coatings, and their therapeutic effects on TBI.

### 3.1. PLGA Nanoparticles

Some researchers have formulated PLGA nanoparticles using poly(lactic-co-glycolic acid), a biocompatible and biodegradable polymer [46,47]. One study utilized PLGA nanoparticles containing fluorescent quantum dots (QD-PLGA), which were observed through live cell imaging after injection into mice via the lateral tail vein. These QD-PLGA nanoparticles did not cross the BBB in the healthy brain of mice but were able to enter the CNS through regions of the BBB that were disrupted [48]. However, NPs that were modified and coated with polysorbate 80 and GSH showed signs of BBB penetration. Research observing PLGAs with S-80 injected into mice showed the highest permeation of BBB and neural uptake of coatings tested. Fluorescence microscopy images demonstrate the accumulation of PS-80 NPs in mice cortices. Historically, the cortex has been a therapeutic target for TBI treatment [49]. PS-80-coated NPs have displayed high efficacy if apolipoprotein E (similar to LDL) is absorbed [50]. Like LDLs, PS-80 NPs coupled with apolipoprotein E are actively taken up by endothelial cells through receptor-mediated endocytosis [51]. These PLGA NPs are also modified by adding polyethylene glycol (PEG) conjugated to 1,2-distearoyl-sn-glycero-3-phosphoethanolamine (DSPE) [52]. Functionalizing the surface with PEG-DSPE has been shown to minimize opsonization through steric hindrance, charge shielding, and prolonging the blood circulation time of the NPs while maintaining stability [53,54]. Additionally, adding glutathione to PEG-DSPE forms glutathione (GSH). GSH has shown promising results because of its transportability in vivo into the BBB via a sodium-dependent transporter [55]. PLGA NPs can be engineered to contain different types of therapeutic molecules to help treat TBI, such as SiRNA and Rolipram. Researchers experimented using an established mouse model of weight-drop-induced TBI [56]. Their results showed that NPs optimally delivered siRNA to the brain early during injuries when the BBB was physically breached and later when the BBB had been self-repaired. These negatively charged siRNA are unable to cross the anionic cell membrane themselves and rely on these NPs to enter and help treat the injury.

### 3.2. Immunomodulatory Nanoparticles

Immunomodulatory nanoparticles (IMPs) can also be used to treat TBI by removing hematogenous monocytes, thereby reducing secondary damage and preserving anatomic and neurologic function. IMPs are negatively charged particles composed of 500 nm biodegradable carboxylated PLGAs. In a previous experiment, these IMPs were infused into wild-type mice that had controlled cortical impact or closed head injury [57]. Once inside, these IMPs bind to the macrophage receptor found on monocytes. These monocytes when bound are no longer sources of inflammation [58]. In both controlled cortical impact and closed head injury, the IMP treatment reduced the number of immune cells that entered the brain, mitigated the inflammation caused by the infiltrating cells, improved long-term motor behavior, and reduced lesion volumes based on anatomic examination.

### 3.3. Leukocyte-Based Biomimetic Nanoparticles

Leukocyte-based biomimetic nanoparticles have also been found to help treat TBI. This nanoparticle was designed to mimic the composition of the cell membrane of leukocytes, including both their lipid and protein components [59]. These NPs were set to target leukocyte inflammation and had the necessary leukocyte membrane protein markers: CD11b, CD19, CD45, and CD47. Cd11b and CD18 are subunits of the CD11 receptor, which is found in monocytes, macrophages, activated microglia, and other immune cells; thus, these protein markers help enhance the targeting of cells that cause neuroinflammation [60]. The CD45 and CD47 markers protect the NPs and avoid mononuclear phagocytic system uptake [61]. These NPs were found to only reach the brain when the BBB was breached after TBI. A previous experiment examined the impact of these NPs on the quantity of F4/80 membrane proteins, which are found in abundance on macrophages that infiltrate the injured cortex after TBI [62]. NPs showed a decrease in F4/80 positive cells and had a lesion volume reduction of about 28.6%.

### 3.4. TN-APNPs Carrying Tat-NR2b9c

TN-APNPs have recently emerged in the field of treating TBIs to transport the promising peptide Tat-NR2b9c. The ionotropic glutamate receptor N-methyl-D-aspartate (NMDA) plays an important role in the normal function of the central nervous system [63]. PSD-95 organizing proteins can bind and connect these receptors to neurotoxic signaling molecules downstream [64]. The Tat-NR2b9c peptide was shown to help reduce NMDA-mediated excitotoxicity by disrupting the interaction of NMDARs with PSD-95 in non-human primates [49]. This interruption prevents NMDARs from contributing to downstream neurotoxic signaling while also preventing the disruption of synaptic activity or calcium influx. TN-APNPs that carry these peptides can further be engineered for targeted delivery through surface conjugation of CAQK, a four-amino-acid peptide that has a high affinity for the extracellular matrix at the injury site [65]. The areas of the brain injured from TBI are known to have elevated amounts of thrombin, which is also the enzyme that cleaves these TN-APNPs open, releasing the peptide. A research study was done in which mice were given TBI through controlled cortical impact. Using an Elevated Plus Maze test, mice injected intravenously with TN-APNPs carrying Tat-NR2b9c compared to the control had lower levels of anxiety, dysphoria, and aggression, which are symptoms of TBI [66].

## 4. Stroke

### 4.1. Current Management

Stroke is the leading cause of long-term disability and the fifth leading cause of death in the US, with ischemic strokes accounting for approximately 87% of all strokes in the country [67]. Following an ischemic stroke, the brain can lose as many neurons as it typically would over 36 years of aging [68]. Tissue plasminogen activator is often used as a treatment for acute ischemic stroke but is limited by its selective efficacy, short window of therapeutic use, and ensuing hemorrhagic complications [69]. No effective palliative or preventative treatments beyond secondary stroke prevention have been designed as of yet [70]. Moreover, most existing approaches to treating neurological conditions like ischemic stroke are invasive and contribute to post-surgical complications [71], underscoring the need to produce a safe, non-invasive, and effective treatment for strokes. Present studies are focused on designing NPs that can provide diagnostic support in stroke detection and function as a drug delivery system (DDS) to the CNS and ischemic brain.

### 4.2. Nanoparticles as a Drug Delivery System

Ischemic strokes induce inflammation, oxidative damage from elevated levels of ROS, ionic imbalances, apoptosis, and reperfusion injuries, resulting in irreversible damage to neuronal function as well as neuronal death [72,73]. The BBB obstructs the entry of 98% of tested drugs and significantly limits the passage of therapeutic drugs into sites of ischemia in the post-stroke brain [68,74,75,76]. As a result, ongoing studies are directed toward utilizing NPs for the delivery of therapeutic agents such as ROS scavengers to the ischemic brain via penetration of the BBB to alleviate neuroinflammation and oxidative damage [77], given their high lipid solubility, nanoscale size, and their ability to covalently bind, adsorb, or encase, then deliver, therapeutic drugs that cannot normally cross the BBB [78,79] in a specific and timely manner [80]. Most strategies involve conjugating NPs with various macromolecules such as surfactants or ligands to instill them with a physical or chemical property of choice [81]. This facilitates the NP penetration into the BBB via adsorptive-mediated transcytosis [75,78,82] or receptor-mediated transport, enabling targeted delivery to specific ischemic tissues while mitigating off-target side effects [83].

### 4.3. Nanoparticles as Agents of Neurotherapy and Enhanced Imaging

NPs as nanocarriers have been shown to traverse the BBB, decrease infarct volume, and reduce neurological deficits by encasing or binding therapeutic drugs, increasing their circulation time in the blood, and promoting their temporally and spatially controlled release at the ischemic site, following IV injection in MCAO mice models [78]. In one study by Karatas, Hulya et al., the authors designed and injected chitosan nanoparticles containing N-benzyloxycarbonylAsp(OMe)-Glu(OMe)-Val-Asp(OMe)-fluoromethyl ketone (Z-DEVD-FMK), a caspase-3 inhibitor, intravenously in MCAO mice models. Two hours pre- or post-treatment, NPs loaded with Z-DEVD-FMK successfully crossed the BBB and resulted in decreased infarct volume, neurological deficit, and ischemia-induced caspase-3 activity [79]. NPs used for stroke treatment can be derived from artificial or organic sources such as micelles, nanotubular particles, inelastic spherical shells, liposomes, gold NPs, platinum NPs, or polymers [80,81]. NPs with biomimetic properties [75], or modifiers such as ligands and membrane derivations [82,83], have been shown to act as effective ROS scavengers [4,41,69] and agents of inflammation resolution [4,84] while decreasing infarct volume [85], reducing blood loss [86], reducing nerve deficit and edema [64], reducing oxidative stress [79], enhancing thrombolysis [4,82,87], and speeding up the recovery of neurological function in vivo in MCAO mice models [84] as a result of the therapeutic drugs they carry [88,89]. In addition, NPs have proven to be effective agents in improving image contrast and sensitivity in MRI scans for strokes [4]. Toth, Gerda B. et al. used ferumoxytol, an iron oxide nanoparticle, as a contrast agent for MRI scans of the brain and other organs. They found that doses as low as 1 mg/kg administered intravenously not only provided high-resolution MRI scans with improved contrast but that ferumoxytol’s long circulation time allowed for multiple high-resolution scans within 72 h until eventually being cleared by the brain’s innate macrophages and astrocytes [90]. Moreover, iron oxide NPs have been used as a contrast agent for MRI scans toward early detection of neuroinflammation in ischemic stroke [77]. In a study by Hubert, B. et al., superparamagnetic iron oxide (SPIO) NPs were used in murine stroke models and successfully tracked phagocytes at the onset of acute ischemic stroke as a measure of inflammatory response [83]. Lastly, ultra-small superparamagnetic iron oxide NPs have been used as MRI contrast agents by Saleh, A. et al., tracking macrophage recruitment to the ischemic brain in post-stroke neuroinflammation in vivo in clinical settings following IV injection [91]. NPs have been shown to enhance MRI resolution and provide precise anatomical targeting while being cleared easily by the body’s phagocytic cells [92]. Thus, NPs are an effective means of both treatment and diagnostics for strokes owing to their nanoscale size and modifiability.

## 5. Translational Potential of Nanoparticle-Based Therapeutics

While NP-based therapies have shown promise in preclinical studies, their translation to clinical practice has been limited, and few NP formulations have advanced to clinical trials. This can largely be attributed to challenges in scaling up manufacturing processes, ensuring consistent quality and reproducibility, addressing potential immunogenicity and toxicity concerns, navigating complex regulatory requirements, and demonstrating long-term safety and efficacy in diverse patient populations. In the context of neurological diseases, including stroke, TBI, and SAH, certain nanotechnologies have reached early-phase clinical trials. For instance, iron oxide nanoparticles such as ferumoxytol have been evaluated as MRI contrast agents and demonstrated utility in visualizing inflammation in ischemic stroke [93,94,95]. Similarly, lipid-based nanoparticles for brain-targeted drug delivery have been explored in phase I/II trials, focusing on their pharmacokinetics and safety profiles [96,97,98]. Some notable examples include lipid nanoparticles for siRNA delivery, such as patisiran (Onpattro) [99,100], liposomal cytarabine (DepoCyt) for chemotherapy delivery [101,102], doxorubicin for targeting brain tumors [103,104], curcumin for neuroinflammation in Alzheimer’s disease [105], and lipid nanoparticles used in mRNA vaccines [106,107]. Given the potential for nanoparticles to cross the blood–brain barrier and deliver targeted therapies, future research should focus on bridging the gap between preclinical findings and clinical applications. Addressing the challenges such as nanoparticle biocompatibility, immunogenicity, and precise targeting mechanisms will be crucial for their successful integration into therapeutic protocols. Continued advancements in nanoparticle engineering and regulatory pathways are expected to facilitate their adoption in clinical settings over the coming years.

## 6. Conclusions

While NPs have shown promise as both diagnostic tools and therapeutic agents in murine models, further investigation is necessary to inform future clinical trials. In particular, researchers have emphasized the importance of further studying NP distribution and optimizing localization to the brain [64,82], as well as safety and toxicity evaluations [108] prior to considering its use in human patients. Nonetheless, it is evident that NPs have the potential to revolutionize therapeutics for SAH, neurotrauma, and stroke.

## Abbreviations

ATX	astaxanthin
BBB	blood–brain barrier
C-NPs	curcumin nanoparticles
CeNPs	cerium oxide nanoparticles
DSPE	1,2-distearoyl-sn-glycero-3-phosphoethanolamine
EBI	early brain injury
GSH	glutathione
HIF-1a	Hypoxia-Inducible Factor-1 alpha
IMPs	immunomodulatory nanoparticles
NMDA	N-methyl-D-aspartate
NP	nanoparticle
Nrf2	nuclear factor-erythroid 2 p45-related factor 2
PFC	perfluorocarbon
PFOB	Perfluorooctyl-Bromide
PGAs	polyglycolides
PLAs	polylactides
PLGAs	poly(lactide-co-glycolides)
ROS	reactive oxygenated species
SAH	subarachnoid hemorrhage
TBI	traumatic brain injury
VEGF	vascular endothelial growth factor
Z-DEVD-FMK	N-benzyloxycarbonylAsp(OMe)-Glu(OMe)-Val-Asp(OMe)-fluoromethyl ketone
